# Problem Gambling Among Spanish University Students: A Gender Perspective Analysis and Its Public Health Relevance

**DOI:** 10.3390/ijerph23020168

**Published:** 2026-01-28

**Authors:** Juan Andrés Samaniego Gisbert, Raquel Suriá Martínez, Nerea Ibáñez Torres

**Affiliations:** 1 Faculty of Health Sciences, Basic Psychology, Universidad Isabel I, 09003 Burgos, Spain; nerea.iba.torres@gmail.com; 2 Department of Communication and Social Psychology, University of Alicante, 03690 Alicante, Spain; raquel.suria@ua.es

**Keywords:** problem gambling, cognitive distortions, public health, university students, prevention, gender

## Abstract

The present study aimed to analyze the differences in psychopathological symptomatology between men and women who participate in online gambling, as well as to explore the relationship between this symptomatology and different risk profiles. The sample consisted of 382 participants, all university students from a province in Spain, of whom 261 were men (68.3%) and 121 were women (31.7%), with a mean age of 21.8 years (SD = 3.2; range = 18–30 years). Psychopathological symptomatology was assessed using the SAS-45, while gambling risk profiles were determined using an ad hoc questionnaire. The results of the risk profiles were formed by categorizing the SOG-RA Scale scores into non-risk gambler, at-risk gambler, and pathological gambler. The results evidenced that gender and risk profile are determining factors in the manifestation of psychopathological symptoms. It was observed that women tend to internalize their emotional problems, presenting higher levels of depression, anxiety, and interpersonal sensitivity, while men exhibit a greater propensity to externalize their symptoms, manifesting hostility, paranoid ideation, and psychoticism. Furthermore, gamblers with high-risk profiles showed higher scores in both internalizing and externalizing symptoms. Significant correlations were identified between risk profile, psychopathological symptomatology, and cognitive distortions, suggesting the need for comprehensive interventions differentiated by gender. These findings provide valuable information for the design of specific treatments that address the emotional and cognitive needs of problem gamblers, contributing to improving the effectiveness of therapeutic strategies in the context of problem gambling. University gambling is an emerging public health issue with consequences that extend beyond the individual, affecting educational, social, and economic well-being. This study addresses a critical gap by delineating gender-specific psychopathological profiles across gambling risk categories, providing actionable evidence to inform campus-based screening and targeted prevention strategies. The findings underscore the necessity of integrating gender-responsive interventions and upstream measures—such as early detection within student health services and harm-reduction messaging—to effectively mitigate gambling-related harm.

## 1. Introduction

In recent years, the increased availability and accessibility of online betting platforms and online games has raised growing concerns about the development of problematic gambling behaviors, especially among the university population. The expansion of the digital environment has facilitated not only access to betting but also to various forms of online gaming that share dynamics based on chance and monetary rewards. This convergence has blurred the boundaries between entertainment and profit-oriented gambling, increasing the risk of developing behavioral patterns similar to those observed in addictive conduct. In this context, problem gambling, also known as ludopathy or problem gambling, has been recognized by the American Psychiatric Association (APA) as an impulse control disorder in the fifth edition of the Diagnostic and Statistical Manual of Mental Disorders (DSM-5-TR) [1]. This pathology is characterized by a persistent and recurrent pattern of maladaptive gambling behavior, leading to significant impairment in the individual’s personal, social, academic, and occupational areas.

A growing need to bet larger amounts of money to achieve the same excitement is observed, as well as multiple unsuccessful attempts to control, reduce, or stop the behavior. Furthermore, individuals may experience considerable emotional distress when trying to cease the activity [2]. This dynamic generates a dependence that seriously compromises the individual’s mental health, also affecting their daily functioning.

One of the main clinical manifestations of this disorder is psychopathological symptomatology, which is commonly classified into two categories: internalizing and externalizing. Internalizing symptoms refer to those that affect the subject’s emotional state and tend to be invisible to the environment, such as anxiety, depression, and social isolation [3]. In contrast, externalizing symptoms are behavioral in nature and are projected outward, directly impacting the individual’s interpersonal relationships, such as impulsivity, irritability, and participation in risk behaviors [4].

Gender plays a crucial role in gambling symptomatology. Women tend to exhibit higher levels of internalizing symptoms such as anxiety and depression, often intensified by sociocultural pressures like managing multiple social and family roles [5,6,7]. Anxiety is linked to financial stress, fear of being discovered, or gambling consequences [6], and in Spain, 47.1% of university students report high anxiety levels [8]. Depression, characterized by hopelessness, sadness, and guilt after losses or relationship deterioration [2], is also more frequent in women, who internalize guilt associated with gambling [9]; 55.6% of university students report depressive symptoms [8]. Social isolation and weakened support networks further affect women due to societal expectations [5,10]. Additional internalizing symptoms include sleep disturbances and physical health problems such as insomnia, hypertension, gastrointestinal, and cardiovascular issues [11,12].

Men, in contrast, exhibit more externalizing symptoms, including irritability, impulsivity, and risk behaviors such as indebtedness, applying for loans, or illegal activities [13,14,15,16]. These behaviors compromise economic and social stability and are partly driven by social pressure to demonstrate success and competitiveness [7,11]. Impulsivity leads to hasty decisions and repeated gambling [15], and men are more likely to face legal and financial consequences [7].

On the other hand, externalizing symptomatology encompasses behaviors that affect the subject’s immediate environment. Irritability is a common trait among pathological gamblers, especially when they are prevented from gambling or suffer significant losses. This can manifest as verbal or physical aggressiveness, significantly altering family and social relationships [13]. Men tend to show this behavior more frequently, in line with traditional models of masculinity that discourage emotional expression [14].

Impulsivity, defined as the inability to resist immediate impulses, constitutes one of the core characteristics of problem gambling. This trait leads to hasty decisions and a marked difficulty in long-term planning, which favors the repetition of gambling behavior [15]. Again, men tend to show higher levels of impulsivity, exposing themselves more frequently to legal and financial consequences [7].

The pattern of risk behaviors, such as indebtedness, applying for loans, or participation in illegal activities, is also widely documented in the population with problem gambling [16]. These behaviors, in addition to compromising economic stability, often generate a progressive deterioration of the individual’s social environment. After conducting a systematic review, Kuss and Griffiths [11], argue that, in most of the studies analyzed, men are more prone to engage in this type of behavior, partly due to social pressure to demonstrate success and competitiveness. Help-seeking also differs by gender. Women, more affected by emotional symptoms, seek mental health services more frequently [9] but face barriers such as stigma or family responsibilities. Men tend to externalize distress through antisocial behaviors or substance abuse, making problem recognition and treatment access more difficult [6]. These differences are shaped by sociocultural factors: women face pressures related to guilt and multiple responsibilities, while men experience expectations of emotional strength that hinder vulnerability expression [11].

In this context, the development of online gambling has introduced a feature particularly relevant from a gender perspective: the possibility of participating anonymously. This circumstance significantly reduces social exposure and external judgment traditionally associated with gambling, which may facilitate women’s engagement in these activities without directly facing the social pressures, stigma, or negative expectations that have historically accompanied female participation in gambling. Consequently, the digital environment could be promoting a greater involvement of women in online games and betting, which in turn raises growing concern regarding the potential increase in the risk of developing problematic or addictive gambling behaviors in this group.

Within this scenario, certain groups may be especially vulnerable to the impact of online gambling. Among them, young users stand out, as they have broad and constant access to the internet and digital devices, facilitating continuous exposure to online gambling platforms and games. This accessibility, combined with factors specific to this life stage, can increase the likelihood of participation and, potentially, the development of problematic gambling behaviors [8,17].

In this sense, the university population represents a particular demographic group that may differ significantly from the general population in terms of online gambling participation and mental health, due to a combination of contextual, psychological, and social factors. First, university students are in a transitional stage toward adulthood, characterized by gaining autonomy, making independent decisions, and exploring new experiences, which may increase exposure to risky behaviors, including online gambling. Additionally, this stage often coincides with high levels of academic stress and performance pressure, predisposing students to use gambling as an emotional coping mechanism or a temporary escape from anxiety and other tensions [9,17].

Second, technological integration and constant access to mobile devices and the internet facilitate the availability of gambling platforms and digital games, making exposure both frequent and immediate [18,19]. Moreover, social media and digital advertising targeted at young adults create an environment that reinforces the normalization and perceived accessibility of gambling [18,20]. Furthermore, university populations exhibit specific psychosocial characteristics, such as increased sensitivity to peer influence and a search for identity and social acceptance, which may interact with the propensity toward problematic behaviors [19,21,22]. Finally, in terms of mental health, research has shown that young university adults display higher rates of anxiety, depression, and perceived stress compared to the general population, which can further increase vulnerability to developing problematic gambling patterns [13,14].

Within this framework, it is essential that studies on participation in online games and betting among the university population incorporate a gender-differentiated approach. The differences observed between men and women in participation patterns, risk profiles, and the expression of psychopathological symptomatology highlight that gender acts as a key moderating factor in the development and maintenance of problematic gambling behaviors [6,9,21]. Analyzing these behaviors without considering gender-specific characteristics may lead to a partial understanding of the phenomenon and to the implementation of less effective preventive strategies and interventions [6,17,22,23]. Therefore, differentiating between men and women in this type of research allows for the identification of specific risk and protective factors, improves early detection, and facilitates the design of prevention and intervention programs better tailored to the real needs of the university population [23,24].

Consequently, given the clear lack of research analyzing in depth gender differences and psychopathological profiles associated with participation in online games and betting, the general objective of this study is to determine whether there are significant differences in the level of participation between male and female university students, as well as in the associated psychopathological symptomatology. To this end, the following specific objectives are established:

1. To explore and characterize gambling risk profiles based on the variable of participation frequency. To examine how gender and risk profile associated with problem gambling relate to psychopathological symptomatology.
2. To determine whether among students who participate in online games and betting, there are significant differences in psychopathological symptoms and in the risk of developing a problem gambler profile.
3. To explore the relationship between psychopathological symptomatology and the risk profile of gamblers based on their participation in online games and betting.

Based on the objectives and previous literature, the following hypotheses were formulated:

**H1:**  *Higher frequency of participation in online gambling is associated with an increased risk profile, with more frequent players showing higher scores in problem gambling indicators.*

**H2:**  *There are statistically significant gender differences in psychopathological symptomatology, with women showing higher internalizing symptoms (*e.g.*, anxiety, depression, interpersonal sensitivity) and men showing higher externalizing symptoms (*e.g.*, hostility, paranoid ideation, psychoticism).*

**H3:**  *Students with higher risk profiles will present significantly elevated psychopathological symptoms compared to students with no risk or low-risk profiles.*

**H4:**  *Psychopathological symptomatology is positively correlated with gambling risk profiles, such that higher levels of internalizing or externalizing symptoms are associated with a higher likelihood of being classified in a risk or problematic gambler category.*

This line of research seeks not only to understand the magnitude of the phenomenon in the university population but also to propose prevention and intervention strategies tailored to the specific needs of each gender, thus promoting a comprehensive approach sensitive to the differentiated realities of problem gambling.

The recognition of online gambling as a significant public health concern is growing, owing to its widespread negative impact on mental health, academic achievement, and social well-being. This view calls for a multifaceted approach that integrates individual clinical interventions with broader preventive and regulatory measures at institutional levels. Young adults in university settings face heightened vulnerability, exacerbated by constant exposure to gambling platforms and pervasive digital advertising. A critical research gap persists in this context: the lack of studies examining sex-specific variations in psychopathology across the entire spectrum of gambling risk within student populations [9]. Our research directly addresses this shortfall by mapping risk profiles and analyzing distinct patterns of internalizing and externalizing symptoms in men and women. The findings offer practical evidence to inform the development of targeted screening, prevention, and intervention programs in university healthcare systems.

## 2. Materials and Methods

### 2.1. Participants

The present study is based on the analysis of a sample consisting of 382 university students from various higher education institutions in the province of Alicante. Specifically, the research included participants from the University of Alicante, the Miguel Hernández University of Elche, and the National University of Distance Education of Elche, (Spain). This institutional diversity allowed for a broader and more representative view of the university reality within the province, facilitating comparative analysis across different academic contexts and teaching modalities. The sample selection was carried out using intentional non-probabilistic sampling, following inclusion and exclusion criteria that guaranteed homogeneity in terms of age (18 to 25 years) and educational level, while ensuring adequate representation of sex and academic disciplines. The distribution of students by academic program was as follows: Psychology accounted for 25% of the sample (96 students), Computer Engineering represented 20% (76 students), Business Administration comprised 15% (57 students), Health Sciences was the largest group with 30% (115 students), and Arts and Humanities included the remaining 10% (38 students).

Of the 382 participants, 198 were women (51.8%) and 184 were men (48.2%). This equitable distribution allowed for a robust comparative analysis between the sexes, a key aspect for examining potential differences in psychopathological symptomatology and behavioral patterns related to gambling. Furthermore, the participants came from diverse areas of knowledge, providing a cross-sectional view of the academic and personal factors that may influence gambling behavior.

### 2.2. Instruments

To assess the risk of problem gambling, some of the criteria established in the DSM-5 and the ICD [1] were followed, as well as the SOGS-RA scale [25], which indicates risk ranges based on gambling activity (once or a few times a year = non-risk gambler; several times a month or several times a week = at-risk gambler; once a day or multiple times a day = pathological gambler). Positive responses were totaled and classified into different player profiles (0–1 positive responses: non-problem gambler; 2–3 positive responses: at-risk gambler; 4 or more positive responses: problem gambler). This instrument was selected based on widely accepted criteria (DSM-5, ICD-11, and SOGS-RA) with the aim of adapting it to the university context. Its use allows for a reduced response burden for participants, facilitating data collection in an academic setting, while maintaining conceptual validity and reliability for identifying risk patterns and problematic gambling behaviors.

The measurement of psychopathological symptomatology was carried out using the Symptom Assessment-45 (SA-45) questionnaire [19], in its version adapted to Spanish by Sandín et al. [23]. This instrument, an abbreviated and validated version of the Symptom Checklist-90-Revised (SCL-90-R), consists of 45 items distributed across nine specific clinical dimensions: somatization, obsessive–compulsive, interpersonal sensitivity, depression, anxiety, hostility, phobic anxiety, paranoid ideation, and psychoticism.

Participants responded to each item using a five-point Likert scale, ranging from 0, indicating “Not at all,” to 4, indicating “Extremely,” based on the intensity with which the symptom has been experienced in the last week. The cultural and linguistic adaptation of the SA-45 for the Spanish-speaking population has demonstrated its suitability and validity, supported by optimal psychometric properties [11]. The internal consistency assessed by Cronbach’s alpha coefficient yielded adequate values for each dimension: depression (α = 0.78), hostility (α = 0.81), anxiety (α = 0.76), interpersonal sensitivity (α = 0.75), somatization (α = 0.79), psychoticism (α = 0.72), obsessive–compulsive (α = 0.70), phobic anxiety (α = 0.80), and paranoid ideation (α = 0.71).

To examine the construct validity of the instrument, a confirmatory factor analysis (CFA) was conducted using the maximum likelihood estimation method. The proposed model included nine latent dimensions corresponding to the theoretical factors of the instrument: depression, hostility, anxiety, interpersonal sensitivity, somatization, psychoticism, obsessive–compulsive, phobic anxiety, and paranoid ideation. The results indicated a good fit of the model to the data. The fit indices were satisfactory (χ^2^/df = 2.31, CFI = 0.94, TLI = 0.92, RMSEA = 0.054, SRMR = 0.048), meeting the criteria recommended in the literature. All standardized factor loadings were significant (*p* < 0.001) and greater than 0.50, ranging from 0.56 to 0.84, which demonstrates an adequate representation of the items within their respective dimensions. Moreover, the correlations among factors ranged from 0.32 to 0.68, indicating moderate associations that support the relative independence of each construct.

### 2.3. Procedure

Data were collected through the administration of a questionnaire to a group of students selected between January and March 2024. The sample was drawn from various university degree programs at participating institutions. Prior to inviting students to participate in the study, academic coordinators were contacted to post the questionnaire link on the universities’ webpages. The questionnaire was hosted on Google Forms and promoted on campus. The estimated time to complete the questionnaire was approximately 10 min.

Before data collection, the study was submitted to the Ethics Committee of the University of Alicante to ensure compliance with ethical research standards. This process included obtaining informed consent from all participants, ensuring that they were fully aware of the study’s objectives, the procedures involved, and any potential risks or benefits associated with their participation. Measures were also taken to protect the privacy and confidentiality of the data collected. Approval from the Ethics Committee confirms that the study was designed and conducted in a manner that respects the rights and well-being of the participants. Additionally, a commitment was signed stating that the data would be used solely for research purposes, maintaining participant anonymity.

### 2.4. Statistical Analysis

For the first objective, which was to assess the risk of problem gambling and establish groups according to problematic use, some of the criteria established in the DSM-5 and ICD, as well as the SOG-RA scale [24], were followed. These criteria classify risk levels based on gambling frequency: playing once or a few times per year is considered a no-risk player; playing several times per month or several times per week is classified as an at-risk player; and playing once or several times per day is defined as a pathological player. Accordingly, the sum of positive responses was used to assign risk profiles: 0–1 positive responses correspond to a no-problem player, 2–3 positive responses to an at-risk player, and 4 or more positive responses to a problem player.

For the second and third objectives, which aimed to determine whether psychopathological symptomatology varies according to gender and the players’ risk profile, a multivariate linear model (MANOVA) was applied. The dependent variables were the global SA-45 scores, as well as the internalizing dimensions (e.g., anxiety, depression, somatization) and externalizing dimensions (e.g., hostility, impulsivity).

The independent variables included in the model were gender (male or female) and player profile (no risk, at risk, pathological). Age was incorporated as a covariate to control for its possible confounding effect on the variables of interest. The assumptions of MANOVA were verified prior to conducting the analysis. Multivariate normality was assessed using Mardia’s test, which did not indicate significant deviations (multivariate kurtosis = 2.41, *p* = 0.118). The presence of multivariate outliers was examined using Mahalanobis distance, identifying three extreme cases that did not exceed the critical threshold (χ^2^(5) = 20.52, *p* < 0.001) and were therefore retained in the sample, as they did not significantly affect the results. The homogeneity of covariance matrices among groups was tested using Box’s M test, which was not significant (Box’s M = 7.83, *p* = 0.214), supporting the fulfillment of this assumption. Likewise, Levene’s tests for each dependent variable confirmed homogeneity of variances (all *p* > 0.05). Overall, these results indicate that the data adequately met the assumptions required for the application of MANOVA, despite the imbalance in group sizes.

To assess the magnitude of the differences found, Cohen’s d index was used as a measure of effect size. In addition, Pearson’s correlation coefficient was used to examine the relationship between psychopathological dimensions and the different risk profiles.

Finally, once the risk profile variable was transformed into a quantitative variable, Pearson’s correlation coefficient was employed to explore the association between risk profile and psychopathological symptomatology.

All statistical analyses were performed using the SPSS program (version 22.0), which allowed for robust multivariate testing and the identification of relevant associations between the examined variables.

## 3. Results

After transforming the scores to group participation frequency according to risk profile, significant sex differences were observed. As shown in Table 1, men presented a higher proportion of individuals with a risk profile (26.1%) compared to women (41.3%), who, in turn, exhibited a higher proportion of problematic cases (9.1%) than men (5.4%). Conversely, the proportion of participants without risk was higher among men (68.6%) than among women (49.6%). The chi-square analysis confirmed that these differences were statistically significant (χ^2^ = 21.46, *p* < 0.001, Φ = 2.41, *p* < 0.001), indicating a higher risk profile among men and a more problematic profile among women who engage in online gaming and gambling activities.

To analyze the psychopathological symptomatology based on sex and the risk profile associated with pathological gambling, a multivariate analysis of variance (MANOVA) was applied, considering sex, risk profile, and their interaction as independent variables, and the psychopathological dimensions as dependent variables. The results indicated statistically significant multivariate differences in both the sex variable (Wilks’ Lambda = 0.97, F(2,375) = 4.80, *p* < 0.05, ηp^2^ = 0.025) and the risk profile (Wilks’ Lambda = 0.96, F(2,750) = 2.96, *p* < 0.001, ηp^2^ = 0.015), evidencing a relevant effect of both variables on psychopathological symptomatology.

In the subsequent univariate analysis (Table 2), significant differences were observed between men and women in the mean scores of the internalizing dimensions (F [1] = 2.80, *p* < 0.05), where women presented higher levels (M = 1.73, SD = 0.72) compared to men (M = 1.59, SD = 0.57), with a small effect size (d = 0.22). On the other hand, in the externalizing dimensions, men showed higher mean scores (M = 1.37, SD = 0.50) compared to women (M = 1.28, SD = 0.58), with significant differences (F [1] = 1.78, *p* < 0.05) and a small effect size (d = 0.17).

Regarding risk profiles, post hoc analyses revealed statistically significant differences in both dimensions, internalizing (F [1] = 1.78, *p* < 0.05) and externalizing (F [1] = 1.78, *p* < 0.05). In particular, the problematic group exhibited higher scores on internalizing factors (M = 1.84, SD = 0.62) compared to the at-risk group (M = 1.73, SD = 0.72) and the no-risk group, albeit with a small effect size (d = 0.48). Likewise, the at-risk group showed significant differences compared to the no-risk group, with a moderate effect size (d = 0.29). Regarding the externalizing dimensions, both the problematic group (M = 1.38, SD = 0.56) and the at-risk group (M = 1.42, SD = 0.67) showed higher means than the no-risk group (M = 1.29, SD = 0.43), with a small effect size (d = 0.24).

**Figure 1:**
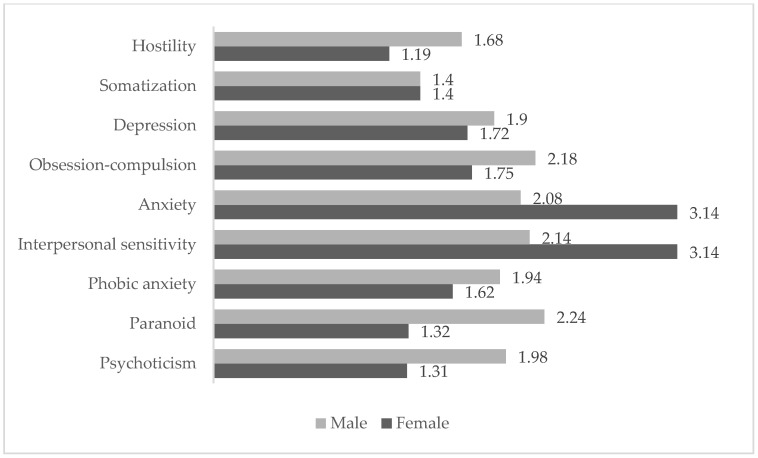
Psychopathological symptoms by sex.

The detailed analysis by sex and risk profile is presented in Figure 1, which shows means and standard deviations for the internalizing and externalizing dimensions in each subgroup.

The specific analysis of the symptoms that make up the internalizing and externalizing dimensions showed significant differences according to sex in most of the indicators evaluated (Figure 1). Men obtained significantly higher scores on externalizing symptoms, notably hostility [t(380) = 5.479; *p* < 0.001; d = 1.20], paranoid ideation [t(380) = 3.618; *p* < 0.001; d = 1.05], and psychoticism [t(380) = 2.644; *p* < 0.001; d = 0.99]. In contrast, women presented significantly higher scores on internalizing symptoms, such as depression [t(380) = −4.262; *p* < 0.001; d = 0.60], anxiety [t(380) = −3.618; *p* < 0.001; d = 0.38], interpersonal sensitivity [t(380) = −4.820; *p* < 0.001; d = 1.48], and phobic anxiety [t(380) = −3.456; *p* < 0.001; d = 0.44].

Spearman’s rank correlation analysis was conducted to examine the associations between risk profile and psychopathological dimensions (Table 3). The results indicated significant positive correlations with somatization (*ρ* = 0.68, *p* < 0.001), psychoticism (*ρ* = 0.61, *p* < 0.001), anxiety (*ρ* = 0.55, *p* < 0.001), paranoid ideation (*ρ* = 0.53, *p* < 0.001), depression (*ρ* = 0.46, *p* < 0.001), phobic anxiety (*ρ* = 0.44, *p* < 0.001), and hostility (*ρ* = 0.24, *p* = 0.003). No significant correlations were found with obsession–compulsion (*ρ* = –0.03, *p* = 0.612) or interpersonal sensitivity (*ρ* = –0.06, *p* = 0.432).

## 4. Discussion

This study makes an original contribution to the literature on online gambling among university populations by addressing three key aspects. First, it provides a specific analysis of Spanish university students, a group that has been less explored compared to clinical populations, allowing for a better understanding of gambling behaviors in a non-clinical but potentially vulnerable demographic. Second, the study systematically applies a gender perspective to gambling behavior and risk perception variables, highlighting how men and women differ not only in participation patterns but also in psychopathological symptomatology. Third, the findings have important public health implications, emphasizing the need for early prevention and intervention strategies within university settings, where students may be particularly exposed to online gambling and its associated risks. By explicitly addressing these aspects, the study adds value to the existing literature and provides a basis for targeted, evidence-based interventions.

Furthermore, this study provides a comprehensive view of the differences in psychopathological symptomatology between men and women, as well as between different risk profiles in online gamblers. The findings highlight the importance of considering sex and risk profile in the development of interventions and treatments for problem gambling, with special emphasis on women and their unique experiences.

In the sample studied, most participants were classified in the no-risk profile (62.6%), while 30.9% fell into the at-risk profile and 6.5% into the problematic profile. These results reflect the heterogeneity of participation patterns, allowing the identification of different levels of vulnerability and guiding preventive strategies according to risk level, in line with previous research on gambling behaviors [6,7]. Accordingly, the findings indicate that a moderate percentage of students present an at-risk gambling profile, consistent with recent studies on university populations in Spain and Europe [6,9]. Likewise, several investigations have highlighted that online gambling is particularly attractive to vulnerable young adults, especially university students, due to the easy access to digital platforms and the normalization of gambling in online environments [6,7,9,17].

When analyzing profiles by gender, significant differences emerged: men showed a higher proportion in the at-risk profile (26.1%), while women had a higher proportion in the problematic profile (9.1%). The chi-square test confirmed a statistically significant association between gender and risk profile. These findings suggest that, although men engage more frequently in risky behaviors, women who participate may experience more severe negative emotional consequences, such as anxiety and depression, which is consistent with recent literature on coping styles and gender differences [9,21,22,23,26].

The MANOVA analysis revealed that both gender and risk profile are significant factors in psychopathological symptomatology, showing that problematic and at-risk players scored significantly higher in both internalizing and externalizing symptoms. These results align with recent evidence indicating that gambling severity is associated with higher psychopathological symptomatology [10,11,24]. The greater tendency of women with risk profiles to internalize emotional problems is supported by research showing that female gamblers more frequently present comorbidity with affective and anxiety disorders, whereas men tend to display a more externalizing and impulsive pattern [3,4,10].

These findings also correspond to recent studies highlighting the interaction between cognitive and emotional factors in the development and maintenance of problematic gambling [15]. Contemporary evidence confirms the central role of gambling-related cognitive distortions—such as the illusion of control, superstitious thinking, and erroneous beliefs about chance—play a central role in reinforcing maladaptive behavior [15,17]. Such distortions contribute to both internalizing symptoms (e.g., frustration and anxiety derived from losses) and externalizing manifestations (e.g., anger and impulsivity), consistent with the current dysfunctional cognition models of gambling [15,17].

Furthermore, recent studies highlight that psychological comorbidity is a key predictor of the transition from recreational to problematic gambling, underscoring the need to to assess psychopathological symptoms and risk profiles together in gambling populations [6,10,15]. Regarding the association between psychopathological symptomatology and player profile, analyses revealed a positive correlation between online gambling frequency and the risk of psychopathological symptoms, particularly in anxiety, depression, and obsessive–compulsive dimensions.

Recent epidemiological research in Spain and Europe reports comsimilar rates of problematic gambling among young adults and university students [5,7,9]. Consistent with these data, several studies indicate that adolescents and young adults may develop behavioral addictions—especially gambling—more quickly than substance addictions, such as alcohol or tobacco [6,9,18]. Sports betting and online gambling are particularly concerning, as they have been identified as major contributors to psychosocial and health-related harms among youth populations [5,6,13,16]. Considering gender is crucial, as differences in risk profiles suggest distinct vulnerability patterns. Women are more likely to internalize emotional problems associated with gambling, while men tend to externalize distress through impulsive or risk-taking behaviors, which is in line with recent findings on coping styles and emotional regulation differences between sexes [3,4,10,23,24,26]. This pattern underscores the importance of gender-sensitive interventions for the prevention and treatment of gambling-related psychopathology, including emotional regulation training, cognitive restructuring of dysfunctional beliefs, and the development of adaptive coping strategies [4,10,22]. In summary, these results reinforce the need for gender-specific interventions, an approach also supported by recent clinical research highlighting sex-related differences in treatment outcomes for problem gambling [4,10,20,26]. These studies suggest that interventions for women should prioritize emotional regulation and psychosocial support, whereas those for men should focus on impulsivity management and behavioral control [4,20]. Moreover, the emphasis on cognitive–behavioral therapy to correct dysfunctional cognitions is consistent with accumulated evidence demonstrating its efficacy in reducing both emotional symptomatology and gambling-related behaviors [19,20,21,22,24].

Despite the significant contributions of this study, several limitations must be considered. First, limitation of the study is related to the use of a shortened and adapted version of the SOGS-RA, DSM-5, and ICD-11 criteria for assessing gambling risk. Although this approach reduces the response burden and is appropriate for the university context, it may limit the depth of assessment and omit certain dimensions of gambling behavior captured by the full instruments. Consequently, some nuances of risk or problematic gambling patterns might not have been fully detected. Moreover, the cross-sectional design prevents establishing causal relationships between gender, risk profile, and psychopathological symptoms; therefore, it cannot be concluded whether the risk profile causes an increase in symptoms or vice versa. Second, the sample focused exclusively on online gambling players, limiting generalizability to other forms of gambling or populations. Additionally, the use of self-reports may be subject to social desirability bias or limited introspective awareness in some participants. Another limitation of the study lies in the decision to merge online gaming and online gambling into a single category. This choice was made due to the difficulty in clearly distinguishing between both types of activities in practice, as many digital platforms integrate recreational gaming and gambling elements within the same virtual environment. However, this merging may have reduced the precision in differentiating the behavioral and motivational patterns associated with each activity. Future studies should consider analyzing these modalities separately in order to gain a more specific understanding of their distinct characteristics and associated risks. Finally, although important variables such as gender and risk profile were considered, other potential mediating or moderating factors—such as socioeconomic status, family history, or social support—were not included, which could influence the manifestation of psychopathological symptoms and cognitive distortions.

## 5. Conclusions

This study provides a detailed and differentiated understanding of psychopathological symptomatology in online gambling players, highlighting the significant influence of gender and risk profiles. Women tend to internalize their emotional problems, while men more frequently exhibit externalizing symptoms, underscoring the need for specific and tailored interventions. Moreover, the observed relationship between risk profile, psychopathology, and cognitive distortions emphasizes the complexity of problem gambling and the importance of addressing it from an integrative perspective that includes both emotional regulation and cognitive restructuring.

Overall, these findings provide a solid foundation for the development of more effective prevention and treatment programs that are sensitive to individual differences, contributing to improved clinical care in this vulnerable population. The results also have relevant implications for education and health promotion in youth and university contexts, suggesting the need for awareness campaigns that include information on gambling risks, self-control strategies, and stress management.

Preventive programs could benefit from integrating early detection tools to identify at-risk individuals before developing problematic patterns, as well as promoting socio-emotional skills, resilience, and healthy leisure alternatives that reduce dependence on gambling for gratification or emotional escape. Mentorship and peer support initiatives could further facilitate the identification and guidance of at-risk youth, fostering protective social environments against problematic behaviors.

Finally, the findings highlight the importance of considering contextual and sociocultural factors, such as academic pressure, exposure to online gambling promotions, and gender norms, which allow for designing more realistic and contextualized interventions. In this way, not only is problematic gambling reduced, but overall well-being is strengthened, and the prevention of emotional and behavioral comorbidities is promoted.

## Figures and Tables

**Table 1:** Risk profiles according to sex.

Sex	No Risk *n*(%)	At Risk *n*(%)	Problematic *n*(%)	Total
Men	179 (68.6%)	68 (26.1%)	14 (5.4%)	261 (100.0%)
Women	60 (49.6%)	50 (41.3%)	11 (9.1%)	121 (100.0%)
Total	239 (62.6%)	118 (30.9%)	25 (6.5%)	382 (100.0%)

**Table 2:** Means, standard deviations, and effect sizes for internalizing and externalizing dimensions by gender and risk profile.

Group	Dimensions	Internalizing M (SD)	F	*p*	d	Externalizing M (SD)	F	*p*	d
Gender									
	Male	1.59 (0.57)	2.80	<0.05	0.22	1.37 (0.50)	1.78	<0.05	0.17
	Female	1.73 (0.72)				1.28 (0.58)			
Risk Profile									
	No risk	1.56 (0.56)	1.78	<0.05	0.29–0.48	1.29 (0.43)	1.78	<0.05	0.24
	At risk	1.74 (0.70)				1.42 (0.67)			
	Problematic	1.84 (0.69)				1.38 (0.56)			

**Table 3:** Spearman’s rank correlations between risk profile and psychopathological dimensions.

Variable	Spearman’s ρ	*p*-Value
Hostility	0.24	0.003
Somatization	0.68	<0.001
Depression	0.46	<0.001
Obsession–Compulsion	–0.03	0.612
Anxiety	0.55	<0.001
Interpersonal Sensitivity	–0.06	0.432
Phobic Anxiety	0.44	<0.001
Paranoid Ideation	0.53	<0.001
Psychoticism	0.61	<0.001

Note: Correlation is significant at the 0.001 level; Correlation is significant at the 0.05 level.

## Data Availability

No new data were created and data is unavailable due to privacy or ethical restrictions.
